# Small bowel volvulus with intussusception: an unusual revelation of neuroendocrine tumor

**DOI:** 10.11604/pamj.2015.22.6.7132

**Published:** 2015-09-03

**Authors:** Imad Lachhab, Boubacar Zan Traoré, Omar Saoud, Yahia Zain Al Abidine Khedid, Fouad Zouaidia, Mahjoub Echarrab, Mohamed Rachid Chkoff

**Affiliations:** 1 Departement of Visceral Surgical Emergency, IBN Sina University Hospital, Rabat, Morocco; 2 Department of Pathology, IBN Sina University Hospital, Rabat, Morocco

**Keywords:** Intestines, small, volvulus, intussusception, neuroendocrine tumor

## Abstract

The primary malignant tumors of the small bowel are rare, representing 1 to 1.4% of all gastrointestinal tumors. We report a case of a 33 year-old women, admitted to our emergency department of visceral surgery for acute abdomen. The clinical examination revealed diffuse abdominal distension, defenseless, the hernia orifices were free and the rectal examination was normal. The biological test showed no hydro electrolytic disorders with normal hemoglobin and normal renal function. The abdominal CT-Scan showed signs of bowel obstruction due to a volvulus with intussusception without ischemia. The patient was operated urgently; the exploration has revealed a small bowel obstruction in the ileum with volvulus, an intussusceptum associated with a retractile mesenteritis, and the hepatic exploration found no metastases. The patient underwent a bowel resection taking away the intussusceptum with the infiltrated mesentery. The postoperative course was uneventful. The pathological result has proved a well-differentiated neuroendocrine tumor with five free nodes. Through this observation, we aim to highlight that an obstruction of small bowel with volvulus and intussusception could be exceptionally due to a neuroendocrine tumor, this complication has enabled a relatively early diagnosis in the absence of metastases and a 6-month follow-up without recurrence is a demonstration.

## Introduction

Primary malignant tumors of the small bowel are rare, representing 1 to 1.4% of all gastrointestinal tumors [1]. The malignant tumors of the small bowel symptoms are not specific; consequently, diagnosis is difficult and delayed. Unlike children, 90% of intussusception in adults are due to a well-established pathology as carcinoma, polyp, Meckel's diverticulum, and benign tumors [2]. Surgical emergency is often secondary to bowel obstruction, perforation and hemorrhage [3]. Through this observation, we aim to highlight that an obstruction of small bowel with volvulus and intussusception could be exceptionally due to a neuroendocrine tumor, this complication has enabled a relatively early diagnosis in the absence of metastases and a 6-month follow up without recurrence is a demonstration.

## Patient and observation

**Figure 1:**
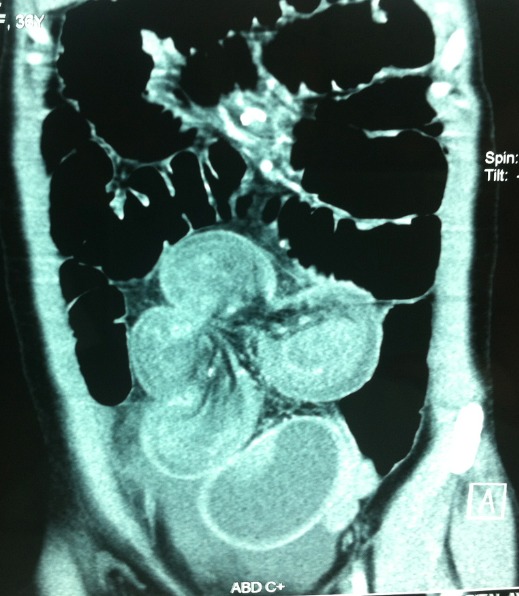
Abdominal CT- Scan, frontal slice, showing the intussusception and the volvulus of the small bowel

A 33 year-old women, of Nigerian origin, was admitted to our emergency department of visceral surgery, with an acute abdomen with a previous history of 3 days of vomiting, pain and abdominal distention without a family history of gastrointestinal tumors. The physical examination revealed a defenseless, diffuse abdominal distension, the hernial orifices were free and the rectal examination was normal. The abdomen without preparation showed air-fluid levels of small bowel type. The biological test showed a WBC at 15200/ml, CRP at 188 mg /L, hemoglobin at 12, 1 g/dl without hydro electrolytic troubles. The abdominal CT-Scan showed bowel obstruction signs due to a volvulus intussusception without ischemia (Figure 1).

**Figure 2:**
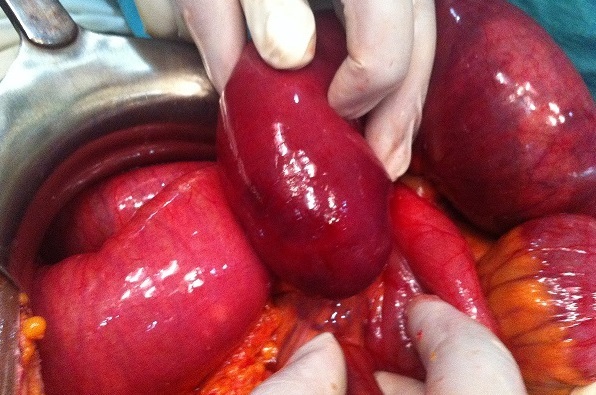
Intussusception and the volvulus of the small bowel

**Figure 3:**
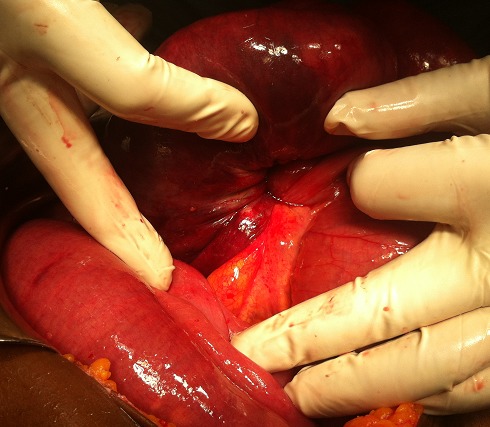
Distal ileum after detorsion showing the retractable mesenteritis

**Figure 4:**
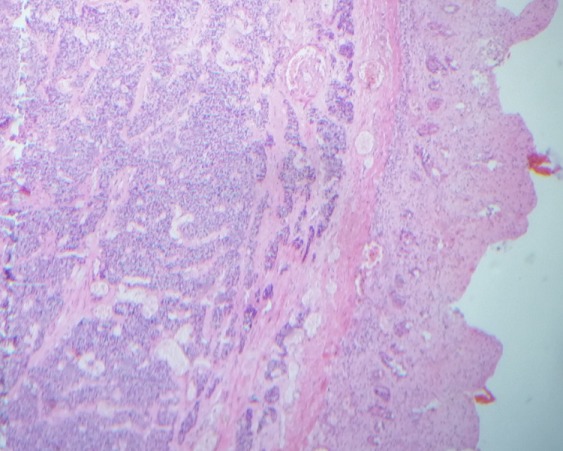
Neuroendocrine tumor of the small bowel showing necrotic wall with hemalun eosin (magnification×100)

**Figure 5:**
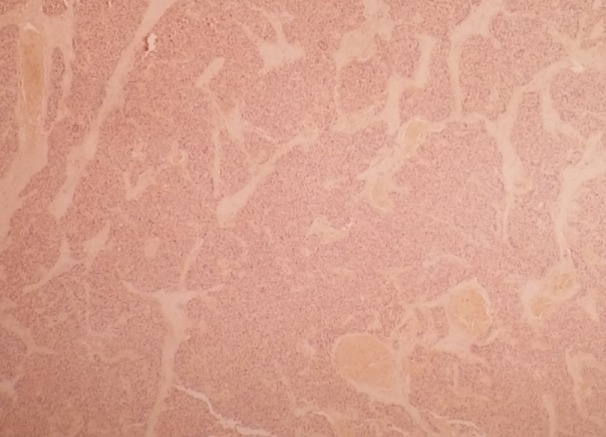
Intense immunohistochimical expression of chromogranine A in small bowel neuroendocrine tumor (magnification×400)

The patient was operated urgently, the exploration revealed a small bowel obstruction in the ileum with volvulus, an intussusceptum (Figure 2) associated to a retractile mesenteritis without ischemia (Figure 3). The liver palpation found no metastasis. The exploration of the rest of the abdomen was normal. The patient underwent a bowel resection taking away the intussusceptum with the infiltrated mesentery. The postoperative course was uneventful. Given the absence of clinical carcinoid syndrome and the absence of metastases, no additional treatment was proposed to the patient. The pathological result has proved a neuroendocrine tumor with free five nodes. The tumor was largely necrotic (Figure 4). A panel of antibodies was performed for the immunohistochemical study, the tumor cells expressed antichromogranine (Figure 5) but they are negative for antisynaptophysine, anti CD 56, and Ki 67. After 6 months of the intervention, the patient was seen in consultation with no complication.

## Discussion

Neuroendocrine tumors are more common in women than in men with a maximum difference between the two genders for the age group between 15 and 19 years (sex ratio = 2.14) and constitute 55% of all endocrine tumors and 12 to 35% of all the small bowel tumors; a family risk for this disease is also reported [4, 5]. Within the gastrointestinal tract, most carcinoid tumors occurred in the small intestine (41,8%), rectum (27,4%), and stomach (8,7%) [6]. Most neuroendocrine tumors are clinically asymptomatic, for this reason they are frequently found incidentally during surgery for potential complications, in endoscopy or in imaging performed for other pathology. The classic triad of carcinoid syndrome makes facial flushing, diarrhea and bronchospasm, sign a metastatic disease almost always hepatic or in case of primary tumor on the systemic circulation (bronchi) and reveals the diagnosis in 2-5% of cases [7–9]. When the neuroendocrine tumor infiltrates the entire wall of the small bowel it creates a desmoplastic reaction followed by obstruction and stenosis [10]. Recently neuroendocrine tumors are diagnosed increasingly and precociously improving the prognosis. The reason for this striking increase of diagnosis is often correlate with the increased use of high-resolution imaging, endoscopy and the improvement of immunohistochemical techniques [11]. In the United States, according to the SEER registry (surveillance, epidemiology and end result), the 5-year survival rate in patients with neuroendocrine tumors of the small bowel has grown up to 51.9% between 1970 s and 1980s and to 60, 5% in 1990′s [6]. STROSBERG et al observed a 5- year survival rate of 75% [12].

Although the mitotic index, female gender, the presence of a carcinoid syndrome is recognized as predictive factors of poor prognosis, tumor size, muscolaris propria invasion, lymph node infiltration or metastases are the safest features to predict the outcomes [13]. Surgery remains a treatment of choice for early neuroendocrine tumors of the jejunum and ileum, peritoneal carcinomatosis or systemic extension characterizes the palliative situation and requires a multidisciplinary approach, especially in the presence of a well-differentiated tumor of the ileum. The treatment of non-metastatic forms based on a complete surgical excision to obtain a resection passing macroscopically to normal area (R0) the only way to improve patient survival at 5 years [6, 7, 9]. Medical treatment appears to be particularly effective if treatment of hepatic metastases is performed [12]. When a carcinoid syndrome appears, the somatostatin analogs are the treatment of choice to control the symptoms. These are initially effective in 60-90% of patients, but can gradually become ineffective. However, treatment with somatostatin analogues is symptomatic treatment and rarely gives a decrease in tumor size [14]. The described case seems to be the third reported case in which the diagnosis was made due to the onset of a volvulus, with only two other similar cases reported in the literature [15, 16]. This complication has allowed a relatively early diagnosis in the absence of metastases and a 6-month follow-up with no recurrence was demonstrated.

## Conclusion

In recent years, major improvements have been made in the diagnosis and in the multidisciplinary approach in the treatment of neuroendocrine tumors of the small bowel, improving the prognosis.
